# Extensive lymphatic spread of papillary thyroid microcarcinoma is associated with an increase in expression of genes involved in epithelial‐mesenchymal transition and cancer stem cell‐like properties

**DOI:** 10.1002/cam4.2544

**Published:** 2019-09-09

**Authors:** Sohee Lee, Ja Seong Bae, Chan Kwon Jung, Woong Youn Chung

**Affiliations:** ^1^ Department of Surgery Eunpyeong St. Mary's Hospital College of Medicine The Catholic University of Korea Seoul Republic of Korea; ^2^ Department of Surgery Seoul St. Mary's Hospital College of Medicine The Catholic University of Korea Seoul Republic of Korea; ^3^ Department of Hospital Pathology Seoul St. Mary's Hospital College of Medicine The Catholic University of Korea Seoul Republic of Korea; ^4^ Department of Surgery Yonsei University Health System Seoul Republic of Korea

**Keywords:** cancer stem cell, epithelial‐mesenchymal transition, lateral neck‐node metastasis, lymphatic spread, thyroid papillary microcarcinoma

## Abstract

**Background:**

Active surveillance is an alternative management for patents with low‐risk papillary thyroid microcarcinoma (PTMC); however, there is an absence of specific molecular markers that predict its progression. We compared gene expression patterns between PTMC with lateral neck‐node metastasis (N1b) and PTMC‐lacking nodal metastasis (N0).

**Methods:**

We performed oligonucleotide microarray analysis in three PTMCs without cervical lymph‐node metastases (N0), and five PTMCs with lateral neck‐node metastasis (N1b) at initial diagnosis, using an Illumina HumanHT‐12 v4.0 Expression BeadChip. Quantitative real‐time PCR (qPCR) and western blot analysis confirmed microarray data. We performed immunohistochemistry (IHC) to confirm protein overexpression in samples from 20 N0 and 24 N1b PTMC patients who underwent thyroidectomy.

**Results:**

Microarray analyses identified 52 probes corresponding to 45 genes. Expression of these genes differed significantly between the two PTMC groups. Forty genes were significantly upregulated and five genes were downregulated in N1b PTMC compared to N0. Four genes related to epithelial‐to‐mesenchymal transition (EMT) and stem cell markers, including ALDH1A3, TM4SF1, PROM1, and CAV1 were significantly upregulated in N1b PTMCs. Real‐time qPCR confirmed this expression and western blot analysis confirmed higher expression of ALDH1A3, TM4SF1, PROM1, and CAV1 in N1b than in N0 PTMCs. IHC indicated overexpression of ALDH1A3 and CAV1 in N1b compared to N0 PTMCs.

**Conclusions:**

Genes related to EMT and thyroid cancer stem cell‐like properties are upregulated in early extensive lymphatic spread of PTMC.

## Graphical abstract

Active surveillance is an alternative management for patents with low‐risk papillary thyroid microcarcinoma (PTMC); however, there is an absence of specific molecular markers that predict its progression. In this study, genes related to EMT and thyroid cancer stem cell‐like properties are upregulated in early extensive lymphatic spread of PTMC.

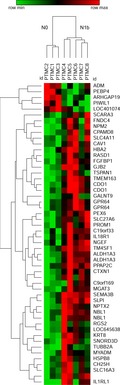

## 1 INTRODUCTION

Small papillary thyroid carcinoma detection is rapidly increasing due to widespread health screening, which has spurred debate about overdiagnosis and overtreatment.^1^, ^2^ Papillary thyroid microcarcinoma (PTMC) is typically indolent and defined as a papillary thyroid carcinoma smaller than ≤ 1.0 cm.^3^, ^4^ Miyauchi et al proposed a trial observation instead of immediate surgery for PTMC patients, and the 2015 American Thyroid Association (ATA) guidelines accept active surveillance as an alternative management option for patents with low‐risk PTMC.^5^, ^6^ However, PTMC with high‐risk features, such as clinical node metastasis, distant metastasis, and suspicion of high‐grade malignancy on cytology, are not suitable for active surveillance and are recommended for immediate surgery.^5^, ^7^, ^8^, ^9^, ^10^ In addition, those with signs of progression during observation should undergo a rescue surgery.^7^, ^9^

Regular ultrasonography has been the only method to identify progressing PTMCs.^7^ Although the Ki‐67 labeling index and a combination of *BRAF* ^V600E^ mutation and *TERT* promoter mutations were described as probable markers, no definite molecular markers can predict whether a PTMC will progress.^7^, ^9^

Here we studied novel molecular markers related to PTMC lateral neck‐node metastasis through epithelial‐mesenchymal transition (EMT) and cancer stem cell properties. We used oligonucleotide microarray analysis and functionally validated these findings.

## 2 MATERIALS AND METHODS

### 2.1 Ethics statement

This study was approved by the institutional review board of the Yonsei University Health System (YUHS), Severance Hospital (4‐2011‐0212), and the Catholic University of Korea, St. Mary's Hospital, Seoul, South Korea (KC18SNSI0691, KC18SESI0229). (http://www.ClinicalTrials.gov Identifier: NCT01384669).

### 2.2 Study subjects and tissue samples

We obtained matched thyroid tumor and normal tissues from eight PTMC patients who underwent thyroidectomy between May 2011 and August 2012, after PTMC diagnosis at the Department of Surgery of YUHS. Of eight PTMC, three did not have nodal involvement and extrathyroidal extension (T1aN0). The remaining five patients had lateral neck‐node metastasis at initial diagnosis (T1aN1b or T3N1b) and underwent modified radical neck dissection combined with thyroidectomy. Immediately after thyroidectomy, we obtained the three pairs of 0.2 × 0.2 × 0.2‐cm cubes of both cancer and normal thyroid tissue from the surgeon; the samples were snap‐frozen in liquid nitrogen at the operation theater and then stored at −80°C. All PTMC were histologically diagnosed as classic papillary carcinoma, and we excluded nonclassical variants such as follicular variant, tall cell variant, or diffuse sclerosing variant from this study.

### 2.3 Gene expression analysis

We used an Illumina HumanHT‐12 v4.0 Expression BeadChip (Illumina, Inc), which is a direct hybridization assay that targets more than 47 000 human probes. We extracted total RNA using TRIzol (Invitrogen Life Technologies) and purified it using RNeasy columns (Qiagen), according to the manufacturers' protocols. RNA purity and integrity were evaluated by A260 and A260/280 ratios using an ultraviolet spectrophotometer (NanoDrop, ND‐1000) and electrophoresis. We verified total RNA integrity using an Agilent Technologies 2100 Bioanalyzer (Agilent Technologies) with an RNA integrity number value. Total RNA was amplified and purified using the TargetAmp‐Nano labeling kit for Illumina Expression BeadChip (EPICENTRE) to yield biotinylated cRNA, according to the manufacturer's instructions. We quantified cRNA by spectrophotometer after purification. After fragmentation, 750 ng of labeled‐cRNA samples were hybridized to each HumanHT‐12 v4.0 Expression BeadChip for 16‐18 hours at 58°C, according to the manufacturer's instructions. Array signal was detected by Amersham fluorolink streptavidin‐Cy3 (GE Healthcare Bio‐Sciences), following the bead‐array manual. We scanned arrays with an Illumina bead‐array reader confocal scanner, according to the manufacturer's instructions. To identify genes with up‐ or downregulated expression, we determined statistical significance of the differentially expressed genes (DEGs) using a paired *t* test, independent *t* test, and fold‐change filtration. We compared PTMC samples with metastasis to those without metastasis using an independent *t* test and a two‐sided *P* < .05 and median fold‐change cutoff of >2.0. Hierarchical cluster analysis clustered DEG groups that behaved similarly across experiments using complete linkage and Euclidean distance. We performed Gene‐Enrichment and Functional Annotation analysis for significant probe lists using DAVID (http://david.abcc.ncifcrf.gov/home.jsp). All data analysis and DEG visualization were conducted using R 2.15.1 (http://www.r-project.org). We performed statistical analysis of functional profiles using *goProfiles* of an R package.

### 2.4 Data availability

Raw and normalized data from gene expression microarray have been deposited into the freely publicly available NCBI′s Gene Expression Omnibus (GEO) database (accession number GSE129562). Website URL: https://www.ncbi.nlm.nih.gov/geo/query/acc.cgi?acc=GSE129562

### 2.5 *BRAF* ^V600E^ mutation analysis

We extracted genomic DNAs from the eight abovementioned PTMC formalin‐fixed, paraffin‐embedded tissue sections using a QIAmp DNA FFPE tissue kit (Qiagen) according to the manufacturer's instructions. *BRAF* ^V600E^ mutation was detected with the Peptide Nucleic Acid (PNA) Clamp BRAF Mutation detection kit (Panagene) according to the manufacturer's instructions.

### 2.6 Quantitative real‐time PCR

We selected four genes that were related to EMT and cancer stem cell markers based on the DEGs in our microarray data and a review of previous literature. Quantitative real‐time PCR was used to validate the expression levels of *ALDH1A3*, *TM4SF1*, *PROM1*, and *CAV1*. We designed primers with Primer Express Version 3.0 (Applied Biosystems) (Table 1). Real‐time qPCR analysis was performed on an Applied Biosystems Prism 7900 sequence detection system (PE Applied Biosystems, http://www.appliedbiosciences.com) with SYBR Green. Amplification conditions were the same for all primers: 50°C for 2 minutes and 95°C for 10 minutes, followed by 40 cycles of 95°C for 30 seconds and 60°C for 30 seconds, then 72°C for 30 seconds. Glyceraldehyde‐3‐phosphate dehydrogenase (GAPDH) was the internal control standard. Experiments were independently performed in triplicate and qPCR cycle numbers were converted to gene amounts (ng) using an accepted formula.

**Table 1:** The gene specific primer of interest genes

Gene symbol	Primer sequence		Size (bp)
ALDH1A3	F	GCCCGTAACAGAACCAGTGT	96
	R	AGGGAAGCCAAATGTGGTAA	
CAV1	F	TTTGCCCAGAAAGAAGATGG	188
	R	CCCAAAGGCAGAATCACAAT	
PROM1	F	GTCCAGCATGGATGAAACCT	181
	R	GGGAATGCCTACATCTGGAA	
TM4SF1	F	ACAATGCTGCTCATTGTTGTG	183
	R	CCATGTTCCAATGATGCTGA	
GAPDH	F	ATGGGGAAGGTGAAGGTCG	108
	R	GGGGTCATTGATGGCAACAAT	

### 2.7 Western blot analysis

We used western blot analysis to confirm protein overexpression for genes of interest in thyroid cancer tissues. Four PTMC tissue samples were analyzed by both microarray and western blot analysis (one N0 and three N1b), and an additional N0 tissue sample was used for western blot analysis. We prepared protein from tissue samples by homogenization in protein lysis buffer (150 mmol/L sodium chloride, 1% v/v Triton X‐100, 1% w/v sodium deoxycholate, 0.1% w/v SDS, 50 mmol/L Tris‐HCl at pH 7.5, and 2 mmol/L EDTA). Protein concentrations were measured by Bradford assay and using bovine serum albumin as a standard. We solubilized equal aliquots of total protein (50 µg) in sample buffer and separated them by electrophoresis on denaturing SDS‐polyacrylamide gels (5% w/v stacking gel and 12% separating gel). Proteins were then transferred to polyvinylidene difluoride membranes. We blocked membranes with 5% (w/v) nonfat dry milk in Tris‐buffered saline (TBS) containing 0.05% (v/v) Tween‐20 and then incubated them with primary antibodies overnight at 4°C, followed by incubation with horseradish peroxidase‐conjugated secondary antibodies for 2 hours at room temperature. The signal indicating antigen‐antibody complexes was detected with WEST‐SAVE UpTM luminol‐based ECL reagent (ABfrontier). Primary antibodies used were ALDH1A3 (1:100, Abcam), TM4SF1 (1:200, Abcam), PROM1 (1:50, Fitzgerald), and CAV1 (1:200, Abcam).

### 2.8 Immunohistochemistry

We also used IHC to confirm protein overexpression for genes of interest in thyroid cancer tissues. We enrolled 44 PTMC patients who underwent thyroidectomy from January 2011 to February 2014 at the Department of Surgery, St. Mary's Hospital in Seoul. Of these, 24 PTMC cases had lateral neck‐node metastasis (N1b) at initial operation, and the other PTMC cases did not have node metastasis (N0). The 4‐μm‐thick sections of FFPE tissue blocks were dewaxed in xylene and then rehydrated through gradients of ethanol to water. After microwave treatment in antigen‐unmasking solution for 15 minutes, the sections were incubated in 3% hydrogen peroxide for 10 minutes to inactivate endogenous peroxidase activity. Then sections were incubated at 4°C overnight with antibodies. Primary antibodies used were against ALDH1A3 (Abcam), TM4SF1 (Abcam), PROM1 (Fitzgerald), and CAV1 (Abcam). Immunostaining was performed using the Vectastain Universal Quick kit according to the manufacturer's instructions. Peroxidase staining was detected with 3, 3‐diaminobenzidine. We omitted antiserum in the negative control. All IHC were reviewed by S Lee. The IHC score was based on the staining intensity and percentage of positive cells. The staining was scored on a four‐tiered scale as follows: "0" (no staining), "1+" (weak staining), "2+" (moderate staining), and "3+" (strong staining). Tumors negative for ALDH1A3, PROM1, CAV1 expressions (staining score 0) were defined as "negative," whereas weakly, moderately, and strongly stained tissue were taken together as "positive." In the case of TM4SF1, a tumor that was stained moderately and strongly was assessed as “positive.”

## 3 RESULTS

The clinicopathologic characteristics of eight PTMC samples are shown in Table 2. There was no significant difference for mean age and mean tumor size between the two groups (*P* = .523 and *P* = .885, respectively). Of the nodal metastasis cases, one showed a minimal extrathyroidal tumor extension and the other had a bilateral cancer lesion. Of five N1b PTMC samples, four cases had perinodal infiltration in metastatic nodes. Three N1b and two N0 PTMCs had *BRAF* ^V600E^ mutations.

**Table 2:** Clinicopathologic characteristics of eight papillary thyroid microcarcinoma patients

No.	Age	Sex	Tumor size (mm)	Capsular invasion	Multiplicity	Lymph node metastasis		Perinodal infiltration	*BRAF* ^V600E^ mutation^a^	Stage (AJCC 7th)	Lymphocytic thyroiditis
						Central (N)	Lateral (N)				
PTMC1	53	F	8	No	No	0/7			Yes	T1N0M0, I	Yes
PTMC2	40	M	4	No	No	0/5			Yes	T1N0M0, I	No
PTMC3	38	F	10	No	No	0/7			No	T1N0M0, I	No
PTMC4	58	F	10	No	Yes, bilateral	4/6	1/29	Yes	Yes	T1N1bM0, IVA	No
PTMC5	36	M	7	No	No	6/8	5/29	No	Yes	T1N1bM0, I	No
PTMC6	47	M	7	No	No	10/14	4/35	Yes	No	T1N1bM0, IVA	No
PTMC7	62	F	10	Yes	No	2/14	11/44	Yes	Yes	T3N1bM0, IVA	Yes
PTMC8	23	F	8	Yes	No	1/6	2/40	Yes	No	T3N1bM0, I	No

a PNA Clamp method.

### 3.1 Gene expression differences between N0 and N1b PTMC

**Figure 1:**
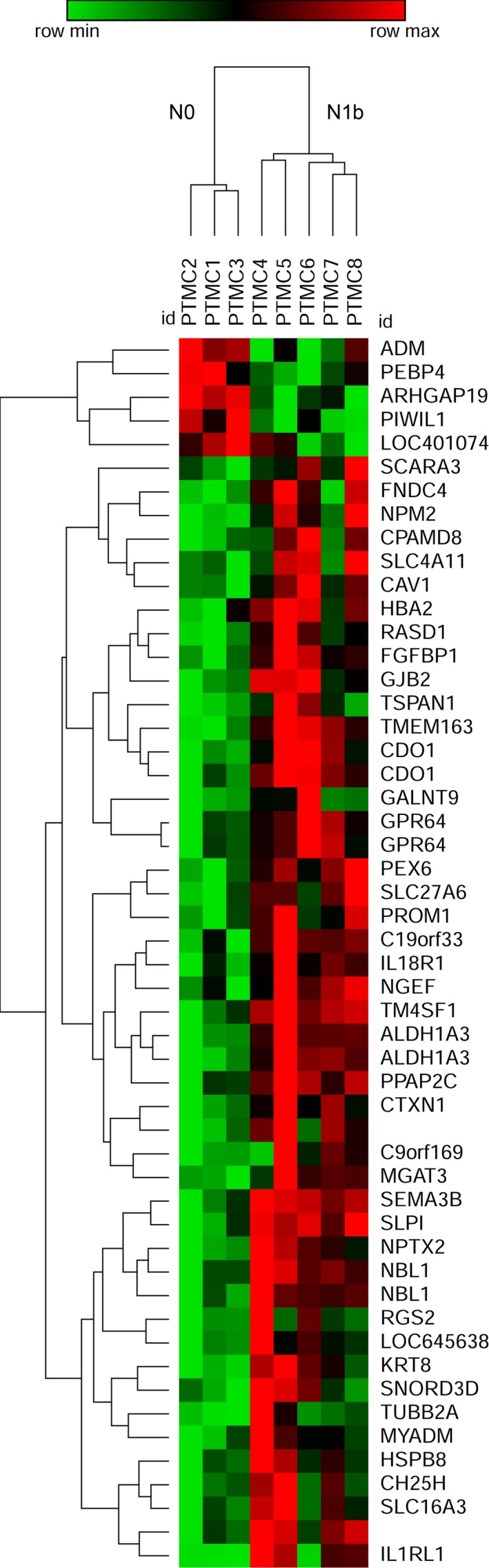
Hierarchial cluster analysis of differentially expressed genes (DEGs) between N0 and N1b PTMC. Red indicates overexpression, green underexpression, and black average expression

We identified 930 probes that corresponded to 798 genes with expressions that differed significantly between PTMC and normal thyroid tissue samples in paired *t* test analysis, using a significance threshold of *P* < .05 with a fold change of ≥2.0. Of these, 413 genes were significantly upregulated, and 385 genes were downregulated in PTMC tissues compared to normal thyroid tissues. The differences in gene expression between N1b and N0 PTMC samples were smaller than those between normal and PTMC samples. We identified 52 probes corresponding to 45 genes that had significantly different expressions between the two groups, using a median fold‐change cutoff of >2.0 and independent *t* test with a *P* < .05 threshold (Table S1). The hierarchical cluster analysis between N1b and N0 PTMC samples found distinct gene expression differences (Figure 1). Of these, 40 genes were significantly upregulated and five were downregulated in N1b PTMC samples compared to N0 samples.

We investigated genes known to be functionally involved with EMT and cancer stem cell properties from the DEGs in N1b PTMC. Four genes, (ie, ALDH1A3, TM4SF1, PROM1, and CAV1) were significantly upregulated in N1b PTMC samples (5.55‐, 3.45‐, 3.11‐, and 2.01‐fold changes, respectively) (Table 3). Of these, ALDH1A3, TM4SF1, and PROM1 were identified as cancer stem cell markers and CAV1 was EMT‐related.

**Table 3:** Epithelial‐to‐mesenchymal transition (EMT) and stem cell marker‐related gene expression in PTMC with lateral neck‐node metastasis vs without node metastasis

Gene symbol	Description	Fold change^a^	*P* value
ALDH1A3	Homo sapiens aldehyde dehydrogenase 1 family, member A3	5.55	.001
TM4SF1	Homo sapiens transmembrane 4 L six family member 1	3.45	.019
PROM1	Homo sapiens prominin 1	3.11	.017
CAV1	Homo sapiens caveolin 1, caveolae protein	2.01	.007

a Fold change of PTMC with lateral neck‐node metastasis/ PTMC without node metastasis.

**Figure 2:**
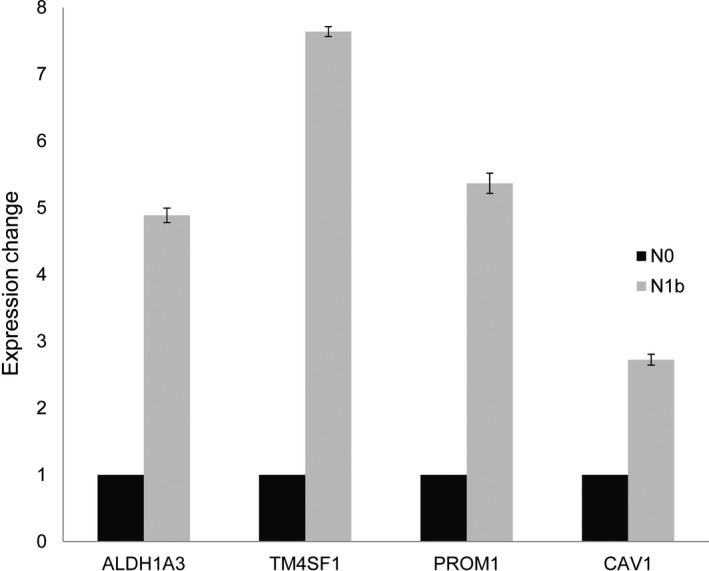
Validation of microarray‐based gene expression by quantitative real‐time PCR. All genes showed significant overexpression in N1b than in N0 (all *P* < .001)

**Figure 3:**
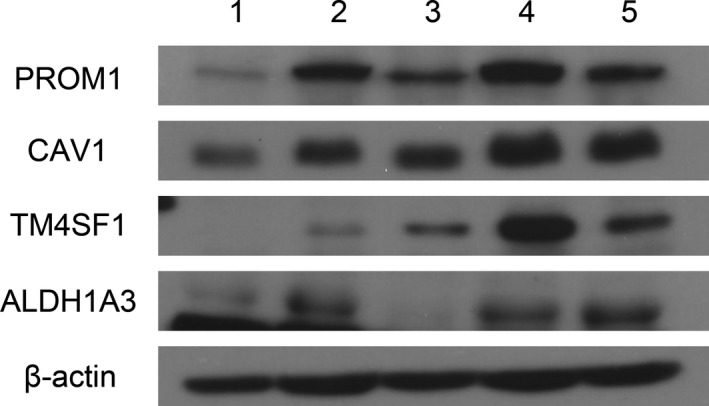
Validation of microarray‐based gene expression by Western blot analysis. Lane numbers 1 and 2 contain N0 PTMC samples; 3, 4 and 5 from N1b PTMCs. Arrows indicate the target band. PROM1, CAV1, and TM4SF1 were more highly increased in N1b PTMC than N0 PTMC. ALDH1A3 showed a weak positivity in N1b PTMC when compared with N1 PTMCs

We supported our findings of DEGs in the microarray data by qPCR (Figure 2). EMT‐related genes including CAV1 showed 2.7‐fold higher expression in N1b PTMC than N0 (all *P* < .001). Additionally, cancer stem cell markers were also overexpressed in N1b PTMC (ALDH1A3: 4.9‐fold, TM4SF1: 7.6‐fold, and PROM1: 5.4‐fold) (all *P* < .001). Western blot analysis showed that protein expression of PROM1, CAV1, and TM4SF1 was higher in N1b PTMC samples than in N0 PTMC samples (Figure 3). ALDH1A3 had a weak positivity in N1b PTMC samples compared to N1 PTMC samples.

### 3.2 Immunohistochemistry

**Figure 4:**
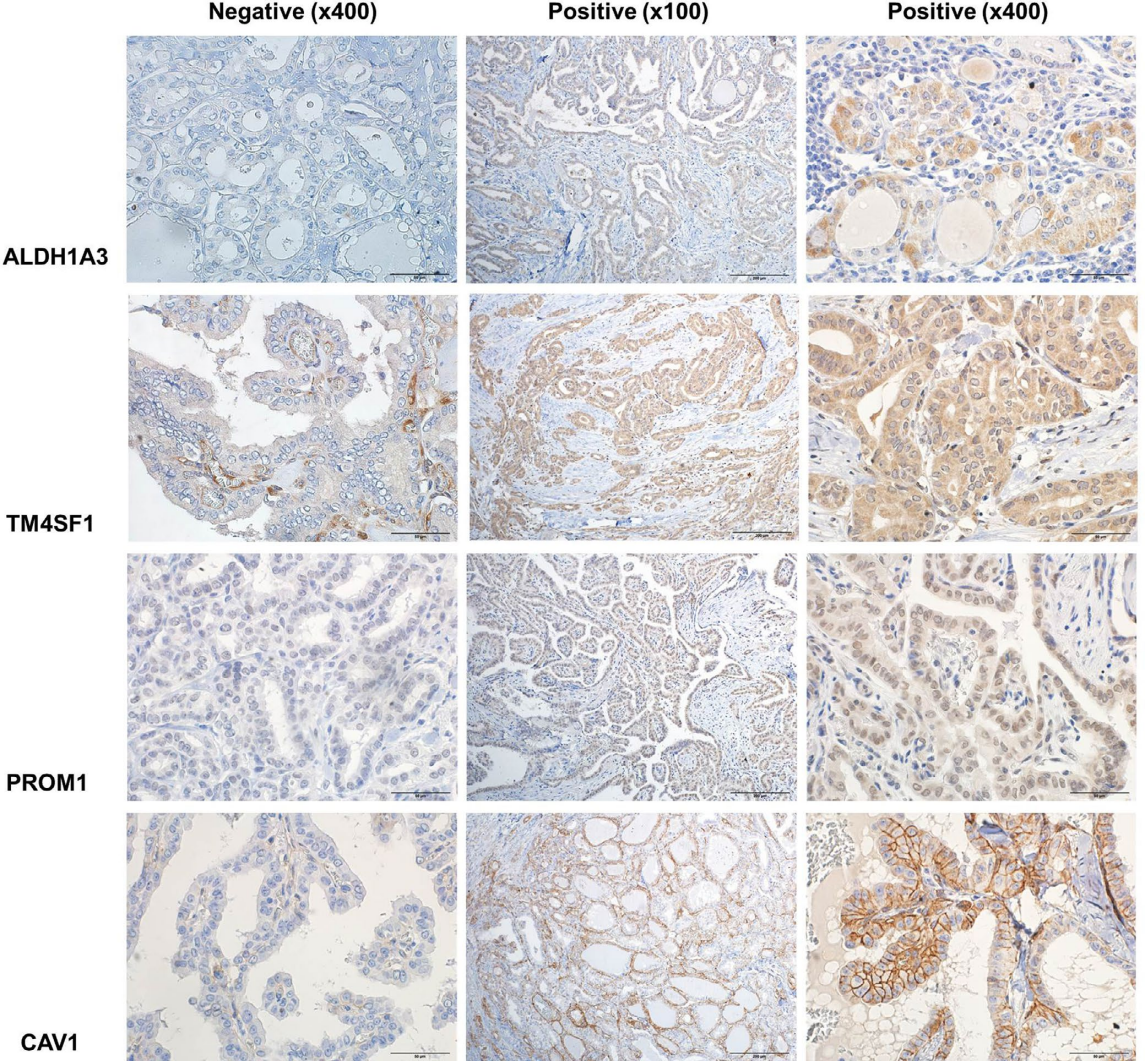
Immunohistochemistry of five proteins in PTMC tissues. The left column (x400) represents negatively stained tissues, and the middle (x100) and right column (x400) represents positively stained tissues. ALDH1A3 and CAV1 were more frequently expressed in N1b; however, expression of PROM1 and TM4SF1 showed no significant differences between the groups

IHC for ALDH1A3, TM4SF1, PROM1, and CAV1 was performed in 44 PTMC samples as an external validation (Table 4 and Figure 4). The mean age, gender ratio, and mean tumor size were not significantly different between the N0 and N1b PTMC groups. Multiple lesion and extrathyroidal extension were found significantly more frequently in the N1b group (*P* < .001). There was no significant difference between vascular and perineural invasion between the groups; however, lymphatic vessel invasion within the tumor was more common in the N1b group (*P* < .001). Regarding the *BRAF* ^V600E^ mutation, we found no statistical difference in its frequency between the two groups. According to IHC results, N1b PTMC samples had more frequent expression of ALDH1A3 and CAV1 (*P* = .003 and *P* < .001, respectively). However, we found no significant difference in the expression of PROM1 and TM4SF1 between the groups.

**Table 4:** Correlation of clinicopathologic features and expression of genes related to epithelial‐to‐mesenchymal transition (EMT) and cancer stem cell markers in 44 patients with PTMC

	N0 PTMC (N = 20)	N1b PTMC (N = 24)	*P*‐value
Age (yr, mean ± SD)	43.1 ± 11.7	43.8 ± 13.7	.858
Gender (M/F)	5/15	7/17	.999
Tumor size (cm, mean ± SD)	0.79 ± 0.10	0.78 ± 0.21	.890
Multiplicity			
No	20 (100%)	10 (41.7%)	<.001
Yes	0 (0%)	14 (58.3%)	
Extrathyroidal extension			
No	20 (100%)	7 (29.2%)	<.001
Yes	0 (0%)	17 (70.8%)	
Vascular invasion			
No	20 (100%)	23 (95.8%)	.999
Yes	0 (0%)	1 (4.2%)	
Lymphatic invasion			
No	19 (95%)	3 (12.5%)	<.001
Yes	1 (5%)	21 (87.5%)	
Perineural invasion			
No	20 (100%)	23 (95.8%)	.999
Yes	0 (0%)	1 (4.2%)	
T stage (AJCC 7th)			
pT1	20 (100%)	7 (29.2%)	<.001
pT3	0 (0%)	17 (70.8%)	
TNM stage (AJCC 7th)			
I	20 (100%)	5 (20.8%)	<.001
IV	0 (0%)	19 (79.2%)	
*BRAF* ^V600E^ mutation			
No	4 (20%)	5 (20.8%)	.999
Yes	16 (80%)	19 (79.2%)	
Immunohistochemistry			
ALDH1A3			
Negative	18 (90%)	10 (45.5%)	.003
Positive	2 (10%)	12 (54.5%)	
CAV1			
Negative	18 (90%)	3 (12.5%)	<.001
Positive	2 (10%)	21 (87.5%)	
PROM1			
Negative	15 (75%)	13 (61.9%)	.505
Positive	5 (25%)	8 (38.1%)	
TM4SF1			
Negative	4 (20%)	5 (21.7%)	.999
Positive	16 (80%)	18 (78.3%)	

## 4 DISCUSSION

Nodal spread of PTC is common; PTMC nodal metastasis occurs in 20%‐56% of cases and early lymphatic spread to the lateral compartment was observed in 4%‐7% PTMC cases at presentation.^11^, ^12^ Various molecular markers are involved in lymph‐node metastasis of PTC; however, no molecular marker can reliably or systematically predict cervical nodal metastasis.^13^, ^14^, ^15^, ^16^ Lymphatic tumor metastasis spreads via lymphatic channels to the draining lymph nodes.^17^ The initial steps of metastasis are thought to include the loss of the epithelial phenotype, gain of mesenchymal morphology, and extravasation to lymphatic and blood vessels.^17^, ^18^ After acquiring an elongated mesenchymal morphology, cancer cells are able to infiltrate surrounding tissues and vasculature, which is followed by metastasis into lymph nodes and distant organs.^17^, ^18^, ^19^ This EMT is proposed to be critical to cancer aggressiveness and invasiveness and is related to the current conceptions of the stem cell‐like properties of cancer cells.^18^, ^19^ Cancer stem‐like cells (CSCs) are a small population within a tumor and have the ability to self‐renew and differentiate into heterogeneous cancer cell lineages.^20^ CSCs are thought to contribute to tumor initiation, systemic dissemination, metastasis, and recurrence after therapy.^19^

In this study, we compared gene expression patterns of N1b and N0 PTMC tissue samples using oligonucleotide microarray hybridization, and then functionally validated our data with qPCR, western blot analysis, and IHC. We found that four DEGs related to EMT and cancer stem cell markers were upregulated in N1b PTMC samples in our microarrays. ALDH1A3, CAV1, PROM1, and TM4SF1 were upregulated according to qPCR and western blot analysis. Additionally, IHC results showed that ALDH1A3 and CAV1 were overexpressed in N1b PTMC.

The aldehyde dehydrogenase (ALDH) is a detoxifying enzyme that acts on oxidized intracellular aldehydes and is highly expressed in various thyroid cancer cell lines.^21^ ALDH is associated with stem‐like properties in various cancers.^21^, ^22^ Shimamura et al reported that ALDH activity could be a major candidate maker for thyroid CSCs.^22^ ALDH‐positive thyroid cancer cells showed higher spherogenicity than ALDH‐negative cells.^22^ Furthermore, when thyroid cancer spheres with high ALDH expression were orthotopically injected into immunocompromized mice, these cancer cells formed tumors and metastases.^23^ Caveolin‐1 (CAV‐1) is a major structural protein in caveolae that participates in cellular transport and signal transduction.^24^ CAV‐1 is involved in epidermal growth factor receptor (EGFR) internalization and CAV‐1 expression is upregulated during EMT.^24^ Janković et al reported thyroid cancer with high‐CAV1/low‐EGFR expression had a more advanced stage and higher degree of neoplastic infiltration than low‐CAV‐1/high‐EGFR.^24^ Paskas et al reported that epithelial expression of CAV‐1 correlated with lymph‐node metastasis and aggressive thyroid cancer.^25^ Prominin‐1 (PROM1), known as CD133, is a major stem cell marker that enhances cancer metastasis.^26^ CD133^+^ cells have CSC properties, including tumorigenicity, metastasis, and radioactive iodine resistance in thyroid cancer cell lines.^26^, ^27^ We found overexpression of PROM1 of N1b PTMC in microarray and confirmed it by real‐time qPCR and western blot analysis. However we found no significant difference in the expression of PROM1 in IHC. Expression of CD133 showed conflicting results in previous reports. Decaussin‐Petrucci et al reported nuclear expression of CD133 that significantly correlated with poor prognosis in young adults, including large tumor size, lymph‐node metastasis, and BRAF mutation.^26^ Jung et al reported that CD133 had diffuse cytoplasmic and membranous uptake in follicular cells and was more frequently expressed in anaplastic thyroid cancer than in papillary thyroid cancer.^28^ Lin et al detected weak expression of CD 133 in normal and lower stage PTC, and an otherwise high level of CD133 expression in stages 3, 4 PTC.^29^ Han et al reported that CD133 did not clinically correlate with aggressive cancer characteristics.^30^ These discrepancies might be due to different staining conditions and cutoff values for positive staining.^26^, ^28^, ^29^, ^30^

Transmembrane 4 L six family member 1 (TM4SF1) is a cell surface low‐molecular weight protein that has four highly hydrophobic transmembrane domains and is highly expressed in human cancer cells.^31^ It is involved in cell migration, invasion, and metastasis.^32^, ^33^, ^34^ Recent studies have examined the correlation of TM4SF1 with microRNAs in tumor angiogenesis of various cancers.^35^, ^36^ TM4SF1 is reportedly one of the candidate markers of thyroid cancer stem cells; however, its clinical application needs to be studied further.^37^ There are no existing scientific studies of TM4SF1 expression in thyroid cancer tissue. In the present study, we defined moderate to strong expression of TM4SF1 as “positive” and found no significant difference in the expression of TM4SF1 between N0 and N1b cases of PTMC. To determine the proper cutoff value for a positive TM4SF1 staining result and its clinical significance, it is necessary to study a larger number of thyroid cancer tissues with various clinical features.

This study had several limitations. First, the sample size of the oligonucleotide microarrays was too small to conclusively identify the key genes impacting early lateral lymphatic metastasis in PTMC. Nonetheless, our study verified the overexpression of candidate genes by IHC using FFPE in an independent patient cohort and showed they were correlated with lateral neck‐node metastasis. A large‐scale study using tissues from prospectively maintained tissue banks will provide accurate information and confirm our findings. Second, to determine the impact of the four genes examined in this study on cervical metastasis of papillary microcarcinoma, the expression levels of these genes should be altered both in vivo and in vitro, and the effects of these alterations (both knockdown and overexpression) should be further examined. Third, it was difficult to determine which specific EMT pathways and cascades are involved in the early lymphatic spread of PTMC in this study. However, evaluation of expression levels of candidate genes in preoperative fine‐needle aspiration cytology may be useful to predict PTMC progression. Additionally, development of therapeutic techniques that regulated or target candidate genes may be helpful to prevent PTMC progression. Therefore, the potential clinical significance of these genes requires further investigation.

## 5 CONCLUSION

Genes related to EMT and thyroid cancer stem cell‐like properties are upregulated during the early extensive lymphatic spread of PTMC. Further evaluation on their clinical significance may be useful in predicting PTMC progression during active surveillance.

## CONFLICT OF INTEREST

The authors declare no conflict of interest.

## AUTHOR CONTRIBUTIONS

S Lee and WY Chung conceptualized and supervised the study, and involved in project administration; S Lee, CK Jung, and WY Chung gave the methodology, and involved in reviewing and editing the draft; S Lee, JS Bae, and CK Jung validated the results; S Lee performed the formal analysis and involved in investigation, original draft preparation and funding acquisition; S Lee, JS Bae, and WY Chung collected the resources and data curation.
