# Supplementary material for: Extensive lymphatic spread of papillary thyroid microcarcinoma is associated with an increase in expression of genes involved in epithelial‐mesenchymal transition and cancer stem cell‐like properties
Source: Cancer Med. 2019 Sep 9;8(15):6528–37. doi: 10.1002/cam4.2544 (PMC6825983; doi:10.1002/cam4.2544)
Supplement: Supplementary file 1 [file CAM4-8-6528-s001.docx]

Supplementary Table 1. Differentially expressed genes in PTMC with and without lateral neck-node metastasis

| GeneSymbol | Definition | Fold-change | *P*-value |
| --- | --- | --- | --- |
| IL1RL1 | Interleukin 1 receptor-like 1 (IL1RL1), transcript variant 2 | 12.1 | 0.019 |
| ALDH1A3 | Aldehyde dehydrogenase 1 family, member A3 (ALDH1A3) | 5.5 | 0.001 |
| CDO1 | Cysteine dioxygenase, type I (CDO1) | 5.1 | 0.007 |
| FGFBP1 | Fibroblast growth factor-binding protein 1 (FGFBP1) | 4.6 | 0.005 |
| RASD1 | RAS, dexamethasone induced 1 (RASD1), | 4.1 | 0.005 |
| TM4SF1 | Transmembrane 4 L six family member 1 (TM4SF1) | 3.5 | 0.019 |
| SLPI | Secretory leukocyte peptidase inhibitor (SLPI) | 3.4 | 0.013 |
| MGAT3 | Mannosyl (beta-1,4-)-glycoprotein beta-1,4-N-acetylglucosaminyltransferase (MGAT3), transcript variant 1 | 3.1 | 0.003 |
| C19orf33 | Chromosome 19 open reading frame 33 (C19orf33) | 3.1 | 0.044 |
| PROM1 | Prominin 1 (PROM1) | 3.1 | 0.018 |
| CPAMD8 | C3 and PZP-like, alpha-2-macroglobulin domain-containing 8 (CPAMD8) | 3.0 | 0.032 |
| TMEM163 | Transmembrane protein 163 (TMEM163) | 2.9 | 0.001 |
| CH25H | Cholesterol 25-hydroxylase (CH25H) | 2.8 | 0.048 |
| GJB2 | Gap junction protein, beta 2, 26 kDa (GJB2) | 2.7 | 0.006 |
| PEX6 | Peroxisomal biogenesis factor 6 (PEX6) | 2.7 | 0.004 |
| SNORD3D | Small nucleolar RNA, C/D box 3D (SNORD3D) | 2.6 | 0.029 |
| SEMA3B | Sema domain, immunoglobulin domain (Ig), short basic domain, secreted, (semaphorin) 3B (SEMA3B), transcript variant 2 | 2.6 | 0.017 |
| SLC27A6 | Solute carrier family 27 (fatty acid transporter), member 6 (SLC27A6), transcript variant 1 | 2.5 | 0.016 |
| NBL1 | Neuroblastoma, suppression of tumorigenicity 1 (NBL1), transcript variant 1 | 2.5 | 0.015 |
| NBL1 | Neuroblastoma, suppression of tumorigenicity 1 (NBL1), transcript variant 2 | 2.4 | 0.009 |
| LOC645638 | misc_RNA (LOC645638) | 2.4 | 0.012 |
| CTXN1 | Cortexin 1 (CTXN1) | 2.4 | 0.006 |
| SLC16A3 | Solute carrier family 16, member 3 (monocarboxylic acid transporter 4) (SLC16A3), transcript variant 2 | 2.4 | 0.038 |
| GPR64 | G protein-coupled receptor 64 (GPR64), transcript variant 4 | 2.4 | 0.028 |
| TUBB2A | Tubulin, beta 2A (TUBB2A) | 2.4 | 0.043 |
| SLC4A11 | Solute carrier family 4, sodium borate transporter, member 11 (SLC4A11) | 2.3 | 0.039 |
| HSPB8 | Heat shock 22 kDa protein 8 (HSPB8) | 2.3 | 0.030 |
| IL18R1 | Interleukin 18 receptor 1 (IL18R1) | 2.3 | 0.037 |
| C9orf169 | Chromosome 9 open reading frame 169 (C9orf169) | 2.2 | 0.040 |
| KRT8 | Keratin 8 (KRT8) | 2.2 | 0.005 |
| GALNT9 | UDP-N-acetyl-alpha-D-galactosamine:polypeptide N-acetylgalactosaminyltransferase 9 (GalNAc-T9) (GALNT9) | 2.2 | 0.048 |
| MYADM | Myeloid-associated differentiation marker (MYADM), transcript variant 2 | 2.2 | 0.029 |
| FNDC4 | Fibronectin type III domain-containing 4 (FNDC4) | 2.1 | 0.028 |
| PPAP2C | Phosphatidic acid phosphatase type 2C (PPAP2C), transcript variant 2 | 2.1 | 0.030 |
| RGS2 | Regulator of G-protein signaling 2, 24 kDa (RGS2) | 2.1 | 0.048 |
| HBA2 | Hemoglobin, alpha 2 (HBA2) | 2.0 | 0.048 |
| SCARA3 | Scavenger receptor class A, member 3 (SCARA3), transcript variant 2 | 2.0 | 0.036 |
| TSPAN1 | Tetraspanin 1 (TSPAN1) | 2.0 | 0.035 |
| CAV1 | Caveolin 1, caveolae protein, 22kDa (CAV1) | 2.0 | 0.007 |
| NPM2 | Nucleophosmin/nucleoplasmin 2 (NPM2) | 2.0 | 0.013 |
| LOC401074 | Hypothetical LOC401074 (LOC401074) | -2.0 | 0.035 |
| PEBP4 | Phosphatidylethanolamine-binding protein 4 (PEBP4) | -2.2 | 0.049 |
| ARHGAP19 | Rho GTPase-activating protein 19 (ARHGAP19) | -2.2 | 0.001 |
| ADM | Adrenomedullin (ADM) | -2.4 | 0.010 |
| PIWIL1 | Piwi-like 1 (*Drosophila*) (PIWIL1) | -2.8 | 0.018 |
