# Supplementary material for: Extensive lymphatic spread of papillary thyroid microcarcinoma is associated with an increase in expression of genes involved in epithelial‐mesenchymal transition and cancer stem cell‐like properties
Source: Cancer Med. 2019 Sep 9;8(15):6528–37. doi: 10.1002/cam4.2544 (PMC6825983; doi:10.1002/cam4.2544)
Supplement: Supplementary file 2 [file CAM4-8-6528-s002.docx]

Supplementary Table 2. Analysis of genes that were differentially expressed between PTMC with and without lateral neck-node metastasis, according to gene ontology results

| System | Gene Category | Adjusted  p value (FDR) | Genes |
| --- | --- | --- | --- |
| GO Biological Process | signaling | 0.001 | PTPRU,GPR64,AGPAT2,NEK6,ADM,LPAR1,EFNA1,F2RL1,TNIK,CLCF1,PPP1R15A,NGEF,TIAM2,GJB2,SDCBP2,TNFRSF21,NRG1,HMOX1,ID1,SMAD3,ATF3,NPTX2,DCDC2,RASD1,RAB23,RGS2,RGS10,RRAD,S100A13,CCL18,VAMP1,TGFA,TGIF1,ZYX,SEMA3B,ARHGAP19,CAV1,IL18R1,IL1RL1,HERPUD1,G3BP2,FGFBP1 |
|  | multicellular organismal process | 0.001 | GPR64,CDO1,NPM2,AGPAT2,RAI2,ADM,CYP1A2,LPAR1,EFNA1,ELF3,EMP1,F2RL1,ALDH1A3,TNIK,CLCF1,NGEF,GJB2,SDCBP2,SLC40A1,NRG1,HMOX1,HPN,ID1,FREM2,SMAD3,NBL1,NPTX2,DCDC2,RAB23,SIX4,PRKX,RGS2,S100A13,SLC11A1,STIM1,VAMP1,TGFA,TGIF1,TUBB2A,SEMA3B,CAV1,PROM1,SLC16A3,IL1RL1,PIWIL1 |
|  | response to stimulus | 0.003 | PTPRU,GPR64,CDO1,AGPAT2,NEK6,ADM,CLDN3,CYP1A2,LPAR1,EFNA1,ELF3,F2RL1,ALDH1A3,TNIK,CLCF1,PPP1R15A,NGEF,TIAM2,HSPB8,SDCBP2,TNFRSF21,CYP2S1,NRG1,HMOX1,HPN,ID1,SMAD3,ATF3,SCARA3,DCDC2,RASD1,RAB23,RGS2,RGS10,RRAD,S100A13,CCL18,NFKBIZ,SLC11A1,STIM1,SULT2B1,TGFA,TGIF1,ZYX,SEMA3B,ARHGAP19,CAV1,IL18R1,SLC16A3,IL1RL1,HERPUD1,G3BP2,FGFBP1 |
|  | developmental process | 0.003 | CDO1,NPM2,AGPAT2,RAI2,NEK6,ADM,CYP1A2,LPAR1,EFNA1,ELF3,EMP1,F2RL1,ALDH1A3,TNIK,CLCF1,NGEF,GJB2,SDCBP2,SLC40A1,NRG1,HMOX1,HPN,ID1,FREM2,SMAD3,NBL1,DCDC2,RAB23,SIX4,PRKX,RGS2,S100A13,TGFA,TGIF1,SEMA3B,CAV1,PROM1,PIWIL1 |
|  | cell proliferation | 0.011 | PTPRU,ADM,EMP1,F2RL1,CLCF1,NRG1,HMOX1,HPN,SMAD3,ATF3,PRKX,S100A13,SLC11A1,TGFA,TGIF1,CAV1,FGFBP1 |
|  | death | 0.016 | PNMA2,LPAR1,ALDH1A3,CLCF1,PPP1R15A,NGEF,TIAM2,HSPB8,TNFRSF21,SLC40A1,HBA2,NRG1,HMOX1,HPN,ID1,SMAD3,SIX4,TGFA,CAV1,HERPUD1 |
|  | locomotion | 0.016 | PTPRU,LPAR1,EFNA1,F2RL1,HMOX1,ID1,SMAD3,DCDC2,SIX4,PRKX,CCL18,SEMA3B,CAV1,SLC16A3 |
|  | biological regulation | 0.029 | PTPRU,GPR64,CDO1,NPM2,AGPAT2,NEK6,PIK3IP1,LMTK3,NCOA7,CYP1A2,LPAR1,EFNA1,ELF3,F2RL1,ALDH1A3,TNIK,CLCF1,PPP1R15A,NGEF,TIAM2,SDCBP2,TNFRSF21,SLC40A1,HBA2,NRG1,HMOX1,HPN,ID1,SMAD3,ATF3,NBL1,DCDC2,RASD1,RAB23,SIX4,PEX6,PDPR,CHPT1,RFX2,RGS2,RGS10,RRAD,S100A13,CCL18,NFKBIZ,SLC11A1,SLPI,STIM1,VAMP1,TGFA,TGIF1,ZYX,PPP1R14C,SLC4A11,ARHGAP19,CAV1,IL18R1,SLC16A3,IL1RL1,PIWIL1,HERPUD1,G3BP2,FGFBP1 |
| GO Cellular Component | extracellular region | <0.001 | ADM,EFNA1,C12orf28,CLCF1,CPAMD8,HCG22,NRG1,HMOX1,FREM2,NBL1,SCARA3,SCUBE2,S100A13,CCL18,SLPI,TGFA,TMPRSS2,SEMA3B,FRAS1,LOXL4,IL1RL1,FGFBP1 |
|  | membrane | <0.001 | PTPRU,TSPAN1,GPR64,AGPAT2,PIK3IP1,LMTK3,CLDN3,CYP1A2,LPAR1,EFNA1,EMP1,F2RL1,NGEF,ABHD12,GJB2,SDCBP2,CPAMD8,TNFRSF21,SLC27A6,CYP2S1,SLC40A1,NRG1,HMOX1,HPN,FREM2,CTXN1,TM4SF1,SMAD3,MGAT3,GALNT9,SCARA3,RASD1,RAB23,PEX6,CHPT1,PLEKHA4,RGS2,RGS10,RRAD,FNDC4,SLC11A1,STIM1,TGFA,TMPRSS2,ZYX,SEMA3B,ELOVL6,C3orf52,FRAS1,RAB11FIP1,TMEM163,PPP1R14C,SLC4A11,LOXL4,CAV1,PPAP2C,IL18R1,PROM1,CH25H,SLC16A3,MYADM,IL1RL1,REEP6,HERPUD1,FGFBP1 |
|  | membrane part | 0.012 | PTPRU,TSPAN1,GPR64,AGPAT2,PIK3IP1,LMTK3,CLDN3,CYP1A2,LPAR1,EFNA1,EMP1,F2RL1,ABHD12,GJB2,TNFRSF21,SLC27A6,CYP2S1,SLC40A1,HPN,FREM2,CTXN1,TM4SF1,FNDC4,SLC11A1,STIM1,VAMP1,TGFA,TMPRSS2,ZYX,ELOVL6,C3orf52,FRAS1,TMEM163,SLC4A11,CAV1,IL18R1,PROM1,CH25H,SLC16A3,MYADM,IL1RL1,REEP6,HERPUD1 |
